# *FUS-DDIT3* Prevents the Development of Adipocytic Precursors in Liposarcoma by Repressing PPARγ and C/EBPα and Activating eIF4E

**DOI:** 10.1371/journal.pone.0002569

**Published:** 2008-07-02

**Authors:** Pedro A. Pérez-Mancera, Camino Bermejo-Rodríguez, Manuel Sánchez-Martín, Fernando Abollo-Jiménez, Belén Pintado, Isidro Sánchez-García

**Affiliations:** 1 Experimental Therapeutics and Translational Oncology Program, Instituto de Biología Molecular y Celular del Cáncer, CSIC/ Universidad de Salamanca, Salamanca, Spain; 2 Department of Medicine, University of Salamanca, Salamanca, Spain; 3 Genetically Engineered Mouse Facility, SEA, University of Salamanca, Salamanca, Spain; 4 Genetically Engineered Mouse Facility, Centro Nacional de Biotecnología (CNB)- Consejo Superior de Investigaciones Científicas (CSIC), Madrid, Spain; Deutsches Krebsforschungszentrum, Germany

## Abstract

**Background:**

FUS-DDIT3 is a chimeric protein generated by the most common chromosomal translocation t(12;16)(q13;p11) linked to liposarcomas, which are characterized by the accumulation of early adipocytic precursors. Current studies indicate that FUS-DDIT3- liposarcoma develops from uncommitted progenitors. However, the precise mechanism whereby FUS-DDIT3 contributes to the differentiation arrest remains to be elucidated.

**Methodology/Principal Findings:**

Here we have characterized the adipocyte regulatory protein network in liposarcomas of FUS-DITT3 transgenic mice and showed that PPARγ2 and C/EBPα expression was altered. Consistent with *in vivo* data, FUS-DDIT3 MEFs and human liposarcoma cell lines showed a similar downregulation of both PPARγ2 and C/EBPα expression. Complementation studies with PPARγ but not C/EBPα rescued the differentiation block in committed adipocytic precursors expressing FUS-DDIT3. Our results further show that FUS-DDIT3 interferes with the control of initiation of translation by upregulation of the eukaryotic translation initiation factors eIF2 and eIF4E both in FUS-DDIT3 mice and human liposarcomas cell lines, explaining the shift towards the truncated p30 isoform of C/EBPα in liposarcomas. Suppression of the FUS-DDIT3 transgene did rescue this adipocyte differentiation block. Moreover, eIF4E was also strongly upregulated in normal adipose tissue of FUS-DDIT3 transgenic mice, suggesting that overexpression of eIF4E may be a primary event in the initiation of liposarcomas. Reporter assays showed FUS-DDIT3 is involved in the upregulation of eIF4E in liposarcomas and that both domains of the fusion protein are required for affecting eIF4E expression.

**Conclusions/Significance:**

Taken together, this study provides evidence of the molecular mechanisms involve in the disruption of normal adipocyte differentiation program in liposarcoma harbouring the chimeric gene *FUS-DDIT3*.

## Introduction

Myxoid/round cell liposarcoma is the most common subtype of liposarcoma, accounting for about 40% of all cases [1], [2]. The tumor cells are characterized by the chromosomal translocation t(12;16)(q13;p11), which produces the *FUS-DDIT3* oncogene [3]–[5]. This oncogene consists of the NH2-terminal domain of *FUS* (previously termed translocated in liposarcoma, *TLS*) fused to the entire codifying sequence of *DDIT3* (previously termed CHOP) [4], [5]. The NH2-terminal domain of FUS confers the transactivation domain to the fusion protein [6], [7]. DDIT3 is a member of the C/EBP family of transcription factors which contains a basic leucine zipper domain and a DNA binding domain, able to form heterodimers with and inactivate other C/EBP members [8]–[12]. *FUS-DDIT3* has not been found in tumor types other than myxoid/round cell liposarcoma [3]–[5].

Early *in vitro* approaches have shown the transforming effects of FUS-DDIT3 in NIH-3T3 fibroblast [13], but not in 3T3-L1 preadipocytes, suggesting that the activity of FUS-DDIT3 was influenced by the cellular environment. Moreover, it has been demonstrated that FUS-DDIT3 blocks the adipogenic potential of NIH-3T3 fibroblast by interfering with the C/EBPβ activity [14]. The ability of FUS-DDIT3 to block adipocyte differentiation is shared, *in vitro*, for DDIT3 in 3T3-L1 preadipocytes [15]–[16], but not in mouse embryonic fibroblasts (MEFs) derived from FUS-DDIT3 and DDIT3 transgenic mice, where FUS-DDIT3, but not DDIT3, is able to block the adipocyte differentiation program in MEFs [17], [18]. However, FUS-DDIT3 shares with DDIT3 the capacity to induce liposarcomas in a xenograft model of human fibrosarcoma cells, suggesting that at least *in vitro*, preadipocytes are not the only target cell of the chromosomal translocation t(12;16)(q13;p11) [19]. Interestingly, FUS-DDIT3 is not able to block adipogenesis in MEFs obtained from aP2-FUS-DDIT3 mice, which express *FUS-DDIT3* under the control of the aP2 promoter, a downstream target of PPARγ expressed in late stages of adipogenesis [20]. Further support to the idea that liposarcoma develops from uncommitted cells comes from the studies showing that the expression of *FUS-DDIT3* in primary mesenchymal progenitor cells give rise to myxoid liposarcoma-like tumors [21], confirming that the cell type is critical for the oncogenic activity of FUS-DDIT3. In agreement with this view is the genomic analysis carried out in human myxoid liposarcoma [22], which is compatible with the genetic program of a primitive target cell from which myxoid liposarcoma could arise. Consistent with this notion, we reported the first *in vivo* evidence for a link between a chimeric protein generated by a chromosomal translocation and a human solid tumor by the generation of transgenic mice expressing *FUS-DDIT3* transgene under the control of the ubiquitous E1Fα promoter, which has found to be functional in mesenchymal progenitor/stem cells [21]. These FUS-DDIT3 transgenic mice developed liposarcomas that resemble their human counterpart [17]. Despite ubiquitously expression of *FUS-DDIT3* oncogene, these mice exclusively developed liposarcomas, suggesting that FUS–DDIT3 may impose an adipocytic program with a partial developmental blockade in mesenchymal cell progenitors. The immature nature of liposarcoma cell progenitors was confirmed by the generation of aP2-FUS-DDIT3 transgenic mice, where FUS-DDIT3, expressed in adipocytes, but not in progenitor cells, is not able to induce liposarcoma development [20]. Moreover, mice expressing the altered form DDIT3–FUS, created by the in-frame fusion of the FUS domain to the carboxy end of DDIT3 also developed liposarcomas [18] indicating that the activity of the fusion protein FUS-DDIT3 is independent of the chimeric junction. By contrast, mice expressing high levels of DDIT3, which lacks the FUS domain, were not able to develop any tumor despite its tumorigenicity *in vitro* [19] although the co-expression of the FUS domain was able to restore liposarcoma development suggesting that it plays a critical role in the pathogenesis of liposarcoma [22]. Taken together, these data indicate that *FUS-DDIT3*-liposarcomas develop from uncommitted progenitor cells in which FUS-DDIT3 prevents the development of adipocytic precursors [23]–[24].

Previous studies have identified a number of transcription factors involved in adipocyte differentiation. These include PPARγ and members of the C/EBP family of transcription factors [25]–[27]. Many of the components of the gene regulatory network that controls the differentiation of adipocytes have been elucidated in studies of cultured 3T3-L1 preadipocytes and MEFs. These transcription factors are expressed as a cascade in which C/EBPβ and C/EBPδ, expressed during the first stages of the adipocyte differentiation program, induce the expression of C/EBPα and PPARγ, the master regulator of adipogenesis. A positive feedback loop mechanism between PPARγ and C/EBPα enhances their activities. This transcriptional cascade finishes with the expression of markers of mature adipocytes such as ap2, adiponectin and adipsin [25]–[27]. There are two PPARγ isoforms generated by alternative splicing, PPARγ1 and PPARγ2, being PPARγ2 more efficient to induce terminal differentiation *in vitro* [28]. In an adipocytic context, the truncated isoforms of C/EBPβ and C/EBPα (LIP and p30, respectively) have a negative effect on adipogenesis, while the full length isoforms (LAP and p42, respectively) enhance the adipocyte differentiation program [28]–[30].

The fact that FUS-DDIT3–associated liposarcomas initiate in uncommitted progenitor cells and generate early adipocytic precursors indicate an important role for *FUS-DDIT3* in the control of early adipocytic development. In this model, the presence of *FUS-DDIT3* would prevent the development of the adipocytic precursors, leading to the observed buildup of the early precursors in liposarcomas [24]. However, little is known about the molecular mechanisms underlying this phenotype. Here, we have unmasked the molecular pathways preventing the development of the adipocytic precursors in liposarcomas induced by the expression of the fusion protein FUS-DDIT3. We demonstrate that FUS-DDIT3 interferes with the PPARγ and C/EBPα activities. In addition, we show that the regulation of the translation machinery by FUS-DDIT3 plays an important role in the blockade of adipogenesis associated to liposarcoma development. The present study establishes for the first time the role of FUS-DDIT3 in preventing the development of adipocytic precursors in liposarcoma.

## Materials and Methods

### Mice

Animals were housed under non-sterile conditions in a conventional animal facility. FUS-DDIT3 mice have been previously described [17]. CombitTA-FUS-DDIT3 mice were generated by cloning the human *FUS-DDIT3* cDNA into the Combi-tTA vector [31]–[33]. Linear DNA fragments for microinjection were obtained by *Not*I digestion and injected into CBA×C57BL/6J fertilized eggs. All experiments were done according to the relevant regulatory standards.

### Histological analysis

Tumor samples were closely examined under the dissecting microscope and processed into paraffin, sectioned and examined histologically. All samples were taken from homogenous and viable portions of the resected sample by the pathologist and fixed within 2–5 min. of excision. Hematoxylin- and eosin-stained sections of each tissue were reviewed by a single pathologist (Teresa Flores). For comparative studies, age-matched mice were used.

### Preparation of primary mouse embryonic fibroblasts (MEFs)

Primary embryonic fibroblasts were harvested from 13.5 d.p.c. embryos and prepared as described [34]. Briefly, head and organs were removed; fetal tissue was rinsed in PBS, minced, and rinsed twice in PBS. Fetal tissue was treated with trypsin/EDTA and incubated for 30 min at 37 °C and subsequently dissociated in medium. After removal of large tissue clamps, the remaining cells were plated out in a 175 cm2 flask. After 48 h, confluent cultures were frozen down. These cells were considered as being passage 1 MEFs. For continuous culturing, MEF cultures were split 1∶3. MEFs and the φNX ecotropic packaging cell line were grown at 37 °C in Dubelcos-modified Eagle's medium (DMEM; Boehringer Ingelheim) supplemented with 10% heat-inactivated FBS (Boehringer Ingelheim). All the cells were negative for mycoplasma (MycoAlertTM Mycoplasma Detection Kit, Cambrex).

### Adipocyte differentiation

Wild-type, FUS-DDIT3 and CombitTA-FUS-DDIT3 MEFs were cultured at 37°C in standard D-MEM:F12 medium (Gibco) supplemented with 10% heat-inactivated FBS (Hyclone), 100 units/ml penicillin (Biowhittaker), and 100 μg/ml streptomycin (Biowhittaker). 10^6^ cells of each genotype were plated to 10 cm plastic dishes and propagated to confluence. Two days after confluence, the adipocyte differentiation program was induced by feeding the cells with standard medium supplemented with 0.5 mM 3-isobutyl-1-Methylxantine (Sigma), 1 μM dexamethasone (Sigma) and 5 μg/ml insulin (Sigma) for two days, and then, with standard medium supplemented with 5 μg/ml insulin for 6 days. This medium was renewed every two days. After 8 days, the appearance of cytoplasmic lipid accumulation was observed by Oil-Red-O staining. Briefly, cells were washed with phosphate-buffered saline (PBS), and then fixed with 3.7% formaldehyde for 2 minutes. After a wash with water, cells were stained with 60% filtered Oil-Red-O stock solution (0.5 g of Oil-Red-O (Sigma) in 100 ml of isopropanol) for 1 hour at room temperature. Finally, cells were washed twice in water and photographed. Lipid accumulation was defined as percentage of cells that are Oil-Red-O positive.

### RNA Extraction

Total RNA from liposarcoma samples were isolated in two steps using TRIzol (Life Technologies, Inc., Grand Island, NY) followed by Rneasy Mini-Kit (Qiagen Inc., Valencia, CA) purification following the manufacturer's RNA Clean-up protocol with the optional Oncolumn Dnase treatment. Total RNA from liposarcoma cell lines was isolated using the Rneasy Mini-Kit (Qiagen Inc., Valencia, CA). The integrity and the quality of RNA were verified by electrophoresis and its concentration measured.

### Reverse Transcription-PCR (RT-PCR)

To analyze expression of *FUS-DDIT3* in human liposarcoma cell lines, CombitTA-FUS-DDIT3 MEFs, and mouse liposarcomas, RT-PCR was performed according to the manufacturer's protocol in a 20-μl reaction containing 50 ng of random hexamers, 3 μg of total RNA, and 200 units of Superscript II RNase H- reverse transcriptase (GIBCO/ BRL). The sequences of the specific primers, which amplifiy specifically the fusion region, were as follows: FUS-F1: 5′-GGTTATGGCAATCAAGACCAG-3′ and DDIT3-B1: 5′-CTTGCAGGTCCTCATACCAGG-3′. The thermocycling parameters for the polymerase chain reaction were as follows: 30 cycles at 94°C for 1 min, 60°C for 1 min and 72°C for 1 min. The PCR products were confirmed by hybridization with specific probes. Amplification of β-actin served as a control to assess the quality of each RNA sample.

### Retroviral infection

FUS-DDIT3 MEFs were infected with high-titers retrovirus stocks produced by transient transfection of φNX cells. The day before the infection, cells were plate at 10^6^ cells per 10-cm dish. Infected MEFs were selected for 3 days with 2 μg/mL of puromycin (Sigma) and replated to carry out the adipocyte differentiation protocol. The mouse PPARγ2 and rat C/EBPα cDNAs were subcloned in the pQCXIP retroviral vector (Clontech).

### Western blot analysis

Whole-cell extracts of exponentially growing cells were prepared in lysis buffer (65 mM Tris pH7, 1% NP40, 2 mM EDTA, 100 mM NaCl) containing the complete cocktail of proteases inhibitors (Roche), and protein concentrations were determined with the Bradford assay reagent (Bio-Rad Laboratories, Inc., Melville, NY, USA). Human adipocyte extract was obtained from Zen-Bio (#TCE-A10-1). Western blot analysis of different cells and tissues were carried out using the Mini Tratans-Blot Cell system (BIO-RAD). Lysates were run on a 10% SDS-PAGE gel and transferred to a PVDF membrane. After blocking in 5% dry milk, the membrane was probed with the following primary antibodies: PPARγ (H-100 and E-8, Santa Cruz Biotechnology), C/EBPβ (C-19, Santa Cruz Biotechnology), C/EBPδ (M-17, Santa Cruz Biotechnology), C/EBPα (14AA, Santa Cruz Biotechnology), FABP4 (aP2) (#10004944, Cayman Chemical), adiponectin (Chemicon International, #MAB3608), eIF2α (Cell Signaling#9722), eIF4E (Cell Signaling#9742) and actin (I-19, Santa Cruz Biotechnology). Reactive bands were detected with an ECL plus system (Amersham).

### Luciferase assays

The reporter containing the proximal part of the hPPARγ2 promoter cloned in front of the luciferase gene (pGL3-hPPARγ2p1000 vector) was kindly provided by Dr. Johan Auwerx (35). The ratC/EBPαwtpSG5 and ratC/EBPβwtpSG5 expression vectors were kindly provided by Dr. Achim Leutz (25). The reporter containing the ratC/EBPα promoter (pCEBP1171) was kindly provided by Dr. Ana Perez-Castillo (36). The expression vectors pcDNA3-hFUS-DDIT3, BOS-hDDIT3 and pcDNA3-NH2-hFUS were generated by cloning the corresponding cDNAs into the expression plasmids. For reporter assays, U2OS cells (human bone osteosarcoma epithelial cells) were transfected using Dual-Luciferase (Promega) with normalization to Renilla luciferase, and mean±standard error was determined from at least three data points. U2OS cells were maintained in DMEM supplemented with 10% fetal bovine serum.

### CAT assays

The CAT reporter containing the ∼2.5 kb proximal promoter region of the murine eIF4E promoter, pm4ECAT, was kindly provided by Dr. Emmett V. Schmidt. C3H10T1/2 cells were maintained in DMEM supplemented with 10% fetal bovine serum. The transfections were carried out using the Profection Mammalia Transfection System Kit (PROMEGA). Cells were harvested ∼60 hr later and extracts were assayed for CAT activity. Relative CAT activities were determined by comparing the ratios of acetylated/unacetylated [14C]chloramphenicol present in spots cut from the thin-layer chromatographs. Equivalent amounts of protein (15–25 mg as determined with Bio-Rad protein kit) and a reaction time of 1 hr were used in all CAT assays, which kept all values within the linear range. Values (average of three independent experiments) show CAT activities relative to extracts of cells transfected with the CAT reporter alone set to a value of 1.

## Results

### Expression of adipogenic genes in liposarcomas arisen in FUS-DDIT3 transgenic mice

**Figure 1:**
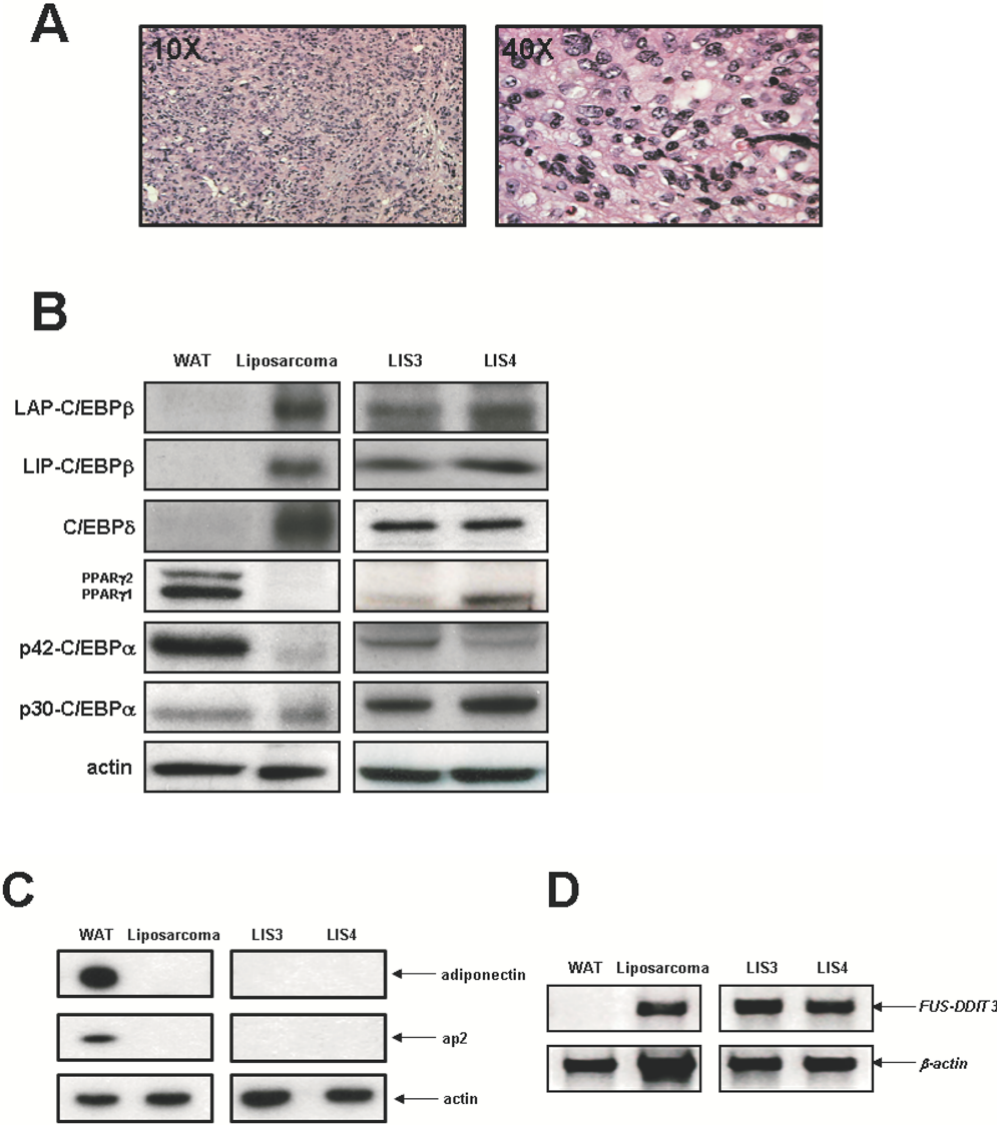
Adipogenic gene expression in liposarcomas of FUS-DDIT3 transgenic mice and in human liposarcoma cell lines carrying the translocation t(12;16)(q13;p11). (A) Hematoxylin/eosin stained sections showing the presence of lipoblasts with round nuclei and accumulation of intracellular lipid in a liposarcoma arisen in the chest region of FUS-DDIT3 mouse (10× and 40× magnifications are shown). (B) Western blot analyses of regulators of adipocyte function in white adipose tissue (WAT), liposarcoma arisen in FUS-DDIT3 transgenic mice and human liposarcomas cell lines expressing FUS-DDIT3 (LIS-3 and LIS-4). Cell and tissue extracts (10 μg) were resolved in SDS-PAGE gel (10% acrylamide), followed by immunoblotting analysis with anti-C/EBPβ, anti-C/EBPδ, anti-PPARγ, anti-C/EBPα and anti-actin antibodies. These data are representative of three independent experiments. (C) Western blot analysis of fat cell markers such as aP2 and adiponectin in liposarcomas of FUS-DDIT3 transgenic mice and in human liposarcoma cell lines carrying the translocation t(12;16)(q13;p11). These data are representative of three independent experiments. (D) Expression of the human *FUS-DDIT3* oncogene by RT-PCR both in liposarcomas of FUS-DDIT3 transgenic mice and in human liposarcoma cell lines carrying the translocation t(12;16)(q13;p11).

The development of adipose tissue involves a differentiation switch that activates a new program of gene expression, followed by accumulation of lipids in a hormone-sensitive manner [25]–[27]. However, liposarcomas are characterized for the accumulation of committed adipocytic precursors named adipoblasts (**Figure 1A**). To explore the molecular basis through which FUS-DDIT3 impairs the normal adipocyte differentiation program, we examined the expression levels of the proteins responsible for normal adipogenesis in liposarcomas arisen in FUS-DDIT3 transgenic mice [17]. Fresh liposarcoma samples were lysed in NP40 lysis buffer and analyzed by western-blot. As shown in **Figure 1B**, the liposarcomas expressed high levels of C/EBPδ and C/EBPβ (LIP and LAP isoforms), which are expressed during the early stages of adipogenesis. On the contrary, liposarcomas expressed low levels of both the transcription factors involved in the late stages of adipogenesis, such as PPARγ1, PPARγ2 and C/EBPα (**Figure 1B**) and the mature adipocyte markers, such as ap2 and adiponectin (**Figure 1C**). The expression of the *FUS-DDIT3* transgene in liposarcomas was assessed by RT-PCR (**Figure 1D**). Interestingly, we found that the truncated C/EBPalpha-p30 isoform was expressed at higher levels than the full length C/EBPalpha-p42 isoform (**Figure 1B**), which is congruent with a blockade in adipocyte differentiation and a transformed phenotype similar to that observed in preadipocyte 3T3-L1 [30]. Taken together, these results suggest that FUS-DDIT3 could prevent adipocytic precursors to differentiate by acting on transcription factors involved in both the early stages of adipogenesis and the late ones.

### Expression pattern of transcription factors governing adipogenesis in human liposarcoma cells

Next, we wanted to confirm that the characteristic expression pattern of transcription factors governing adipogenesis detected in liposarcomas coming from FUS-DDIT3 transgenic mice was also present in human liposarcoma cells. In order to address this aim, we took advantage of two human liposarcoma cell lines harboring the chromosomal translocation t(12;16) and expressing the *FUS-DDIT3* chimeric gene (**Figure 1D**). The analysis of the expression pattern of these transcription factors in both human liposarcoma cell lines confirmed an expression pattern similar to that previously observed in liposarcomas derived from FUS-DDIT3 transgenic mice (**Figure 1B-C**), although we detected variable, although low, levels of PPARγ1 in the human liposarcoma cell lines. These results demonstrate that tumors arisen in the FUS-DDIT3 transgenic mouse mimic human liposarcomas both histologically and molecularly. Taken together, these findings suggest that FUS-DDIT3 could prevent the development of committed adipocytic precursors in liposarcoma through the interference with PPARγ and C/EBPα expression, two transcription factors with a critical role in adipogenesis.

### *In vivo* suppression of FUS-DDIT3 rescues the adipocyte differentiation block

**Figure 2:**
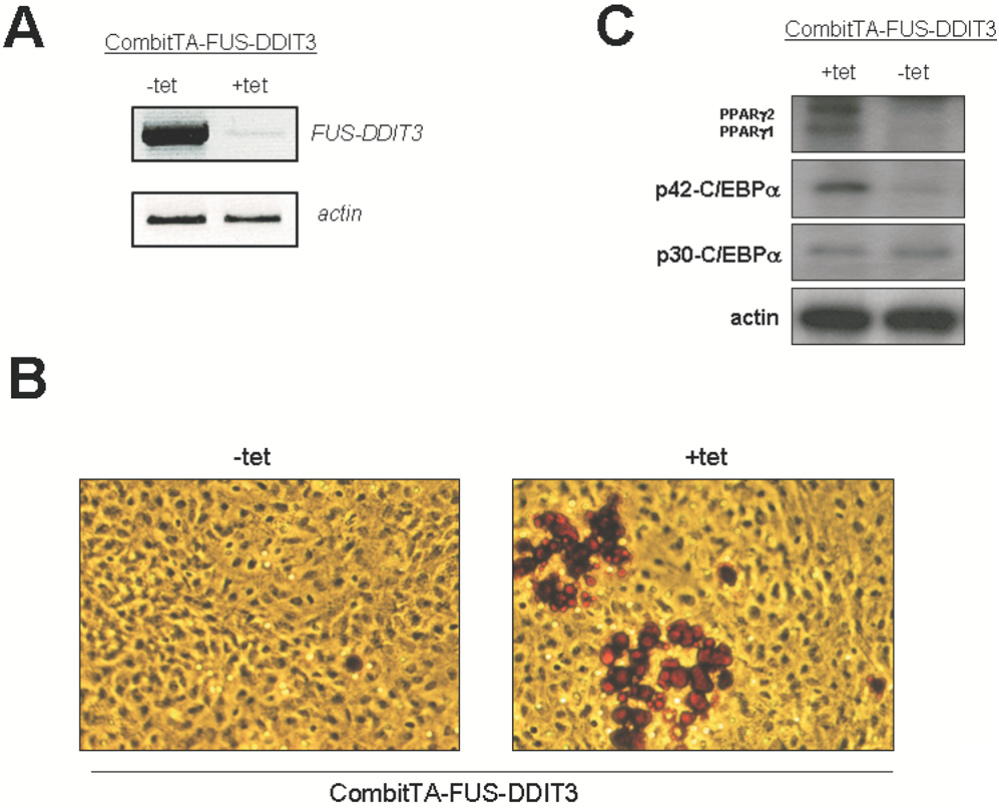
CombitTA-FUS-DDIT3 expression and effect of FUS-DDIT3 on adipocyte differentiaton. A) Analysis of the tetracycline (Doxycycline) dependent CombitTA-FUS-DDIT3 expression by RT-PCR in the presence (+tet) or in the absence (-tet) of doxycycline in MEF (the time of treatment with doxycycline was 48 hours). Actin was used to check the RNA integrity and loading. B) Adipocyte differentiation in CombitTA-FUS-DDIT3 MEFs after suppression of FUS-DDIT3 expression by tetracycline treatment. CombitTA-FUS-DDIT3 MEFs in the presence (+tet) or in the absence (-tet) of doxycycline were cultered up to confluence and grown in the presence of standard adipose differentiation induction medium. At day 8 after induction of adipocyte differentiation, cells were fixed and stained for neutral lipids with Oil-Red-O and the morphological differentiation is shown (the original magnification is ×20). This experiment was repeated three times using cells prepared from different embryos and similar results were obtained. C) Western blot analyses of PPARγ, and C/EBPα in liposarcoma arisen in CombitTA-FUS-DDIT3 mice in the presence (+tet) or in the absence (−tet) of doxycycline. Doxycycline was given at 4 mg/mL for 4 weeks.

The above results support the view that FUS-DDIT3 expression is enough to induce the adipocyte differentiation block. In order to determine this, we generated transgenic mice using the Combi-tTA system, in which the expression of *FUS-DDIT3* gene could be exogenously regulated. This system, which has the transactivator and the tet-operator minimal promoter driving the expression gene unit on a single plasmid [31]–[33], ensures the integration of the transactivator and reporter gene units in equal copy numbers in a direct cis-configuration at the same chromosomal locus and prevents genetic segregation of the control elements during breeding. Insertion of the *FUS-DDIT3* gene under the control of the tetO-minimal promoter yielded the plasmid CombitTA-FUS-DDIT3 and mice were generated. CombitTA-FUS-DDIT3 expression was determined in MEFs after culturing for two days in the presence or absence of tetracycline (**Figure 2A**). CombitTA-FUS-DDIT3 was detected in MEFs without tetracycline but not in cells cultured with tetracycline (20 ng/ml). To further examine the contribution of FUS-DDIT3 to adipogenesis, we isolated MEFs from days 13.5 of CombitTA-FUS-DDIT3 embryos (**Figure 2B**). At day 8 after hormonal induction, there is not lipid accumulation, defined as percentage of cells that are Oil-Red-O positive, in CombitTA-FUS-DDIT3 MEFs (1–3%). However, this differentiation block was reverted upon doxycycline treatment of CombitTA-FUS-DDIT3 MEFs (15–25%) (**Figure 2B**). Similarly, the impaired expression of PPARγ and C/EBPα in liposarcomas of CombitTA-FUS-DDIT3 mice was normalized following administration of tetracycline (4 gr/L in the drinking water for 2 weeks, a dose sufficient to suppress of exogenous -FUS-DDIT3 expression) (**Figure 2C**). The demonstration that FUS-DDIT3 downregulation was sufficient to normalize the adipocyte differentiation capacity of FUS-DDIT3 cells further indicates that PPARγ2 and C/EBPα were regulated directly by FUS-DDIT3.

### The adipogenesis defects in FUS-DDIT3 MEFs can be rescued by ectopic expression of PPARγ2

**Figure 3:**
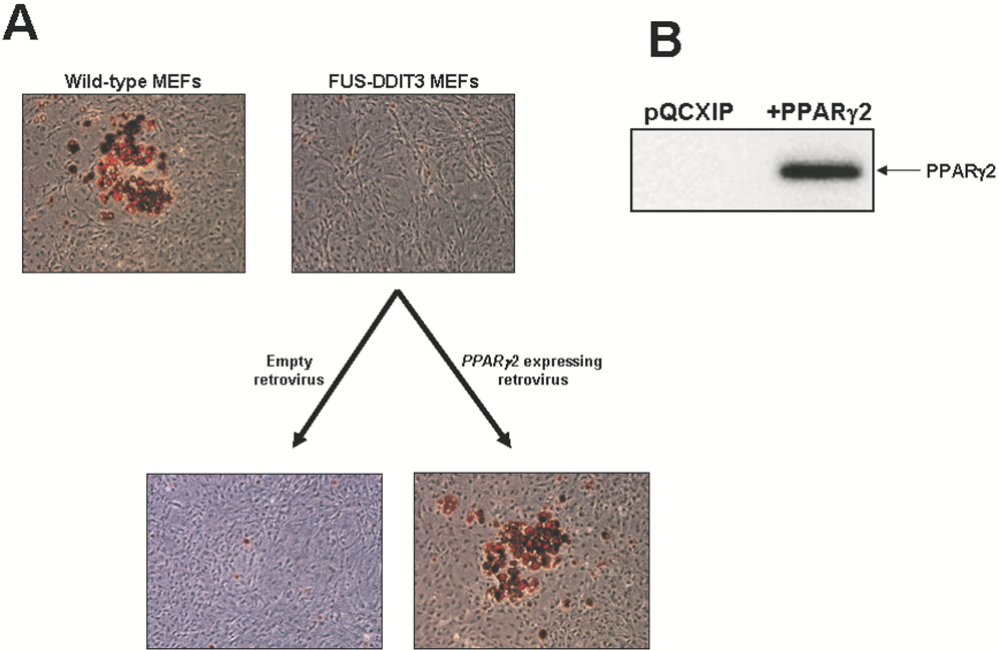
Retroviral-mediated expression of PPARγ2 rescues the impaired adipogenesis of FUS-DDIT3 MEFs. A) FUS-DDIT3 MEFs were infected with either control retroviral vector or one expressing PPARγ2 (pQCXIP-PPARγ2) and selected for 3 days with 2 μg/ml puromycin. Then, wild-type MEF, FUS-DDIT3 MEF and PPARγ2 expressing FUS-DDIT3-MEF were cultered up to confluence and grown in the presence of standard adipose differentiation induction medium. At day 8 after induction of adipocyte differentiation, cells were fixed and stained for neutral lipids with Oil-Red-O and the morphological differentiation is shown (the original magnification is ×20). This experiment was repeated three times using cells prepared from all lines and from different embryos and similar results were obtained. B) Analysis of the PPARγ2 protein by western-blot in FUS-DDIT3 MEFs infected with either a control retroviral vector (pQCXIP) or one expressing PPARγ2 (pQCXIP- PPARγ2) 4 days after infection.

Our data revealed that PPARγ2 expression is modulated by FUS-DDIT3, suggesting an interesting link between this gene and FUS-DDIT3. In order to confirm this transcriptional regulation we re-introduced PPARγ2 in both control and FUS-DDIT3 MEFs by retroviral transduction and evaluated the adipogenesis capacity and the expression level of PPARγ2 by western-blot (**Figure 3**). The adipogenesis of MEFs by hormonal induction is a well established model system for the study of adipocyte differentiation [34], [35]. To further examine the contribution of PPARγ2 to FUS-DDIT3-mediated adipogenesis, we isolated MEFs from days 13.5 of FUS-DDIT3 and control embryos. At day 8 after hormonal induction, there is lipid accumulation in control MEFs (20–35%) and this lipid accumulation is lacking in FUS-DDIT3 MEFs (**Figure 3A**). However, retrovirus-mediated expression of PPARγ2 in FUS-DDIT3-MEFS re-established the adipocyte differentiation capacity to wild-type levels as shown in **Figure 3A**. The demonstration that PPARγ2 was sufficient to normalize the adipocyte differentiation capacity of FUS-DDIT3 cells further indicates that PPARγ2 was regulated directly by FUS-DDIT3 and it plays a critical role in the blockade of the adipocyte differentiation of FUS-DDIT3 adipocytic precursors.

### FUS-DDIT3 represses the PPARγ2 promoter

**Figure 4:**
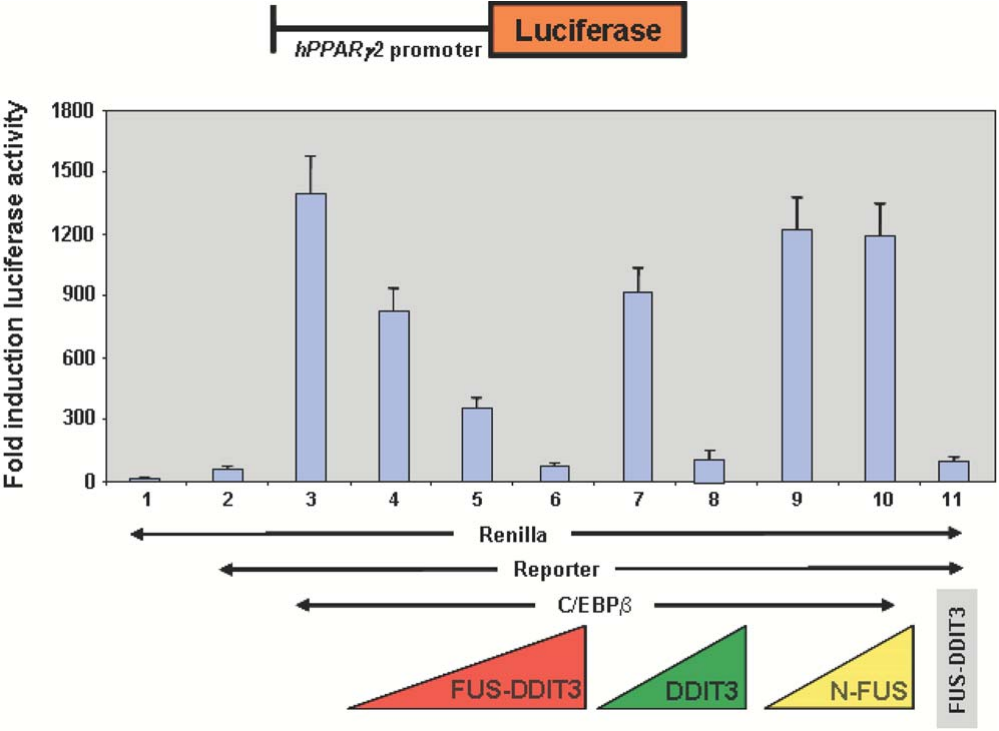
FUS-DDIT3 represses the PPARγ2 promoter. A 1 kb proximal promoter region of human PPARγ2 was previously shown to be sufficient to drive the PPARγ2′s expression in reporter assays [34. 35] and it is active in U2OS cells when co-transfected with C/EBPβ expression vectors. To directly assess the ability of FUS-DDIT3 to modulate transcription from DNA sequences present in the PPARγ2 promoter, an expression vector containing either the human *FUS-DDIT3* cDNA, the human DDIT3 domain or the human FUS domain were co-transfected into U2OS cells along with the reporter vector containing the PPARγ2 promoter (pGL3-hPPARγ2p1000 vector) and C/EBPβ expression vector (ratC/EBPβ wtpSG5). Luciferase reporter assays demonstrate that FUS-DDIT3 repressed the human *PPARg2* reporter in a DDIT3·dependent manner. In all lines, 1 μg of pRL-SV40 (Renilla basal control (PROMEGA) was used for normalization of the results along with 5 μg of pGL3-hPPARγ2p1000 (lines 2–10); 3 μg of ratC/EBPβ wtpSG5 (lines 3–10); 3, 5 and 7 μg of the hFUS-DDIT3 expression vector (lines 4–6, respectively); 3 and 7 μg of the hDDIT3 expression vector (lines 7–8, respectively); 3 and 7 μg the NH2-hFUS expression vector (lines 9–10, respectively); 5 μg of the hFUS-DDIT3 expression vector (line 11). These data are representative of three independent experiments.

Because the results so far suggest that *FUS-DDIT3*directly regulates PPARγ2 expression, we examined whether FUS-DDIT3 might be directly involved in the control of PPARγ2 transcription. A 1 kb proximal promoter region of human PPARγ2 was previously shown to be sufficient to drive the PPARγ2′s expression in reporter assays [34], [35] and it is active in U2OS cells when co-transfected with C/EBPβ expression vector (**Figure 4**). To directly assess the ability of FUS-DDIT3 to activate transcription from DNA sequences present in the PPARγ2 promoter, an expression vector containing a *FUS-DDIT3* cDNA was co-transfected into U2OS cells along with the reporter vector containing the PPARγ2 promoter (pGL3-hPPARγ2p1000 vector) and with C/EBPβ expression vector. Co-expression of *FUS-DDIT3* repressed luciferase activity (**Figure 4**).

Previous results have provided evidence that both the FUS and the DDIT3 domains of FUS-DDIT3 play a specific and critical role in the pathogenesis of liposarcoma [18]. Thus, we next investigated which FUS-DDIT3 domain was responsible for the repression of the PPARγ2 promoter. Using the same system, we showed that while the co-expression of the domain NH2-FUS did not produce any effect on the transactivation capability of C/EBPβ, the co-expression of the DDIT3 domain produced a dramatic repression in the activation of the PPARγ2 promoter by C/EBPβ, indicating that the DDIT3 domain of FUS-DDIT3 was involved in the repression of the PPARγ activity in liposarcomas by interfering with the C/EBPβ activity (**Figure 4**). FUS-DDIT3 might be inhibiting this C/EBPβ transcriptional activity by forming heterodimer as it has been previously shown in NIH-3T3 fibroblasts [14].

### C/EBPα cannot rescue the impaired adipogenesis of FUS-DDIT3 MEFs

**Figure 5:**
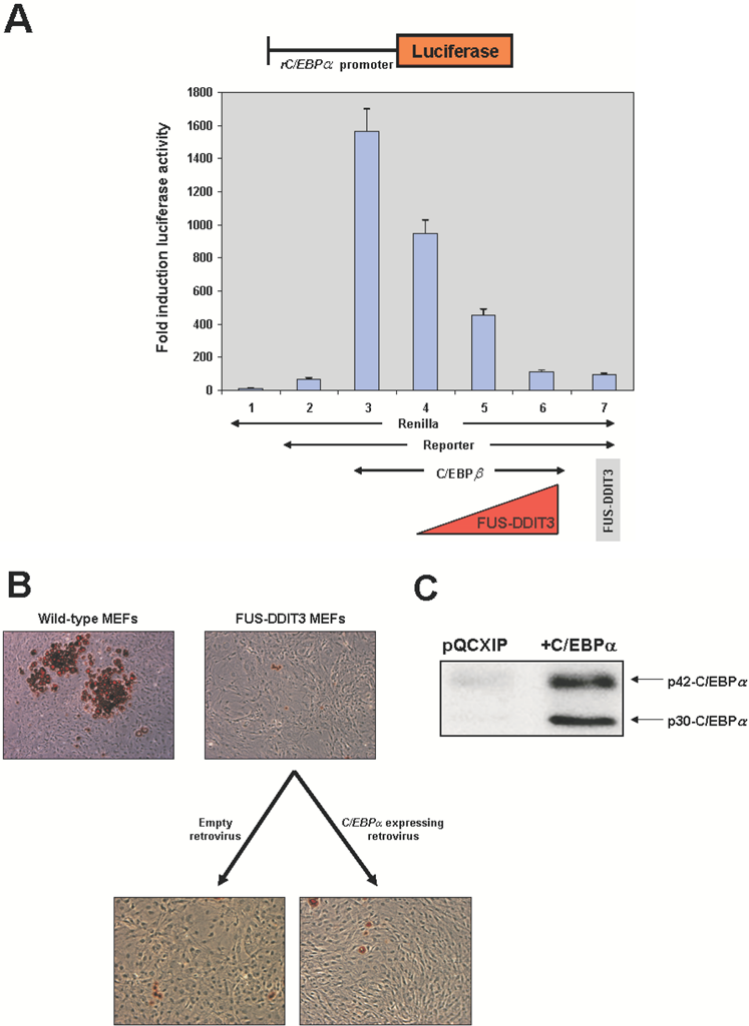
C/EBPα does not bypass adipogenesis blockade in FUS-DDIT3 expressing-MEF. (A) FUS-DDIT3 represses the C/EBPα transactivation induced by C/EBPβ. U2OS cells were cotransfected with 1 μg of pRL-SV40 (Renilla basal control (PROMEGA), lines 1–6) along with: 5 μg of pCEBP1171 (luciferase reporter vector containing 1171 of the rat C/EBPα promoter, samples 2–6); 3 μg ratC/EBPβwtpSG5 (C/EBPβ expressing vector, lines 3–6); 3, 5 and 7 μg pcDNA3-hFUS-DDIT3 (hFUS-DDIT3 expression vector, lines 4–6); 5 μg of the hFUS-DDIT3 expression vector (line 7). These data are representative of three independent experiments. (B) Retroviral expression of C/EBPα does not rescue the adipocyte differentiation blockade in FUS-DDIT3 MEFs. FUS-DDIT3 MEFs were infected with a retroviral vector expressing C/EBPα (pQCXIP-C/EBPα) and selected for 3 days with 2 μg/ml puromycin. Then, wild-type MEF, FUS-DDIT3 MEF and C/EBPα expressing FUS-DDIT3-MEFS were cultered up to confluence and grown in the presence of standard adipose differentiation induction medium. At day 8 after induction of adipocyte differentiation, cells were fixed and stained for neutral lipids with Oil-Red-O and the morphological differentiation is shown (the original magnification is ×20). This experiment was repeated three times using cells prepared from all lines and from different embryos and similar results were obtained. (C) Analysis of C/EBPα (p42-C/EBPα and p30-C/EBPα isoforms) protein expression by western-blot in FUS-DDIT3 MEFs infected with either a control retroviral vector (pQCXIP) or one expressing C/EBPα (pQCXIP- C/EBPα) 4 days after infection.

To further define the molecular mechanism by which FUS-DDIT3 alters the adipogenic potential of MEF, we next addressed how FUS-DDIT3 regulates the expression of C/EBPα. In **Figure 1B** we have previously shown that liposarcomas arisen in FUS-DDIT3 transgenic mice, similarly to humans, expressed lower levels of C/EBPα than wild type WAT, which is associated with an immature phenotype. This observation suggests that *FUS-DDIT3* regulates C/EBPα expression. In order to examine whether FUS-DDIT3 might be directly involved in the control of C/EBPα transcription, we carried out reporter gene assays using a luciferase reporter gene containing 1171 bp of the promoter region of the rat C/EBPα gene (pCEBP1171) [36]. This reporter was previously shown to be sufficient to drive the C/EBPα′s expression in reporter assays [36] and it is active in U2OS cells when co-transfected with C/EBPβ expression vector (**Figure 5A**). To directly assess the ability of FUS-DDIT3 to activate transcription from DNA sequences present in the C/EBPα promoter, an expression vector containing a *FUS-DDIT3* cDNA was co-transfected into U2OS cells along with the reporter vector containing the C/EBPα promoter (pCEBP1171) and with C/EBPβ expression vector. Co-expression of *FUS-DDIT3* repressed luciferase activity (**Figure 5A**). Therefore, FUS-DDIT3 is also the primary responsible for the transcriptional down-regulation of C/EBPα by interfering with C/EBPβ activity.

However, it has been previously reported that ectopic expression of C/EBPα is unable to induce adipogenesis in a PPARγ null background [37], although in contrast, PPARγ restores adipocitic potential in C/EBPα null fibroblast [38]. We have shown that PPARγ2 induces terminal adipocyte differentiation in FUS-DDIT3 expressing MEF (**Figure 3A**). Thus and with the aim of clarifying the relationship between the PPARγ and C/EBPα pathways in liposarcomas, we next assessed the role of the C/EBPα downregulation in the process of adipogenesis in FUS-DDIT3 MEFs, as these MEFs show a dramatic downregulation and inactivation of PPARγ (**Figure 1B**). We investigated whether C/EBPα was also able to overcome the blockade in adipocyte differentiation shown by FUS-DDIT3-MEF. FUS-DDIT3 MEFs were infected with a retroviral vector expressing C/EBPα. To define whether overexpression of C/EBPα could rescue adipogenesis in FUS-DDIT3 cells, adipocytic differentiation was induced in FUS-DDIT3 MEFs infected either with empty vector or with the C/EBPα expressing vector. Of interest, the impaired adipocyte differentiation block in FUS-DDIT3 MEFs was not normalized by restoring C/EBPα at day 8 after hormonal induction, (**Figure 5B**), although the retroviral vector was producing C/EBPα correctly as defined by western-blot (**Figure 5C**).

### FUS-DDIT3 up-regulates expression of eIF4E in liposarcomas

**Figure 6:**
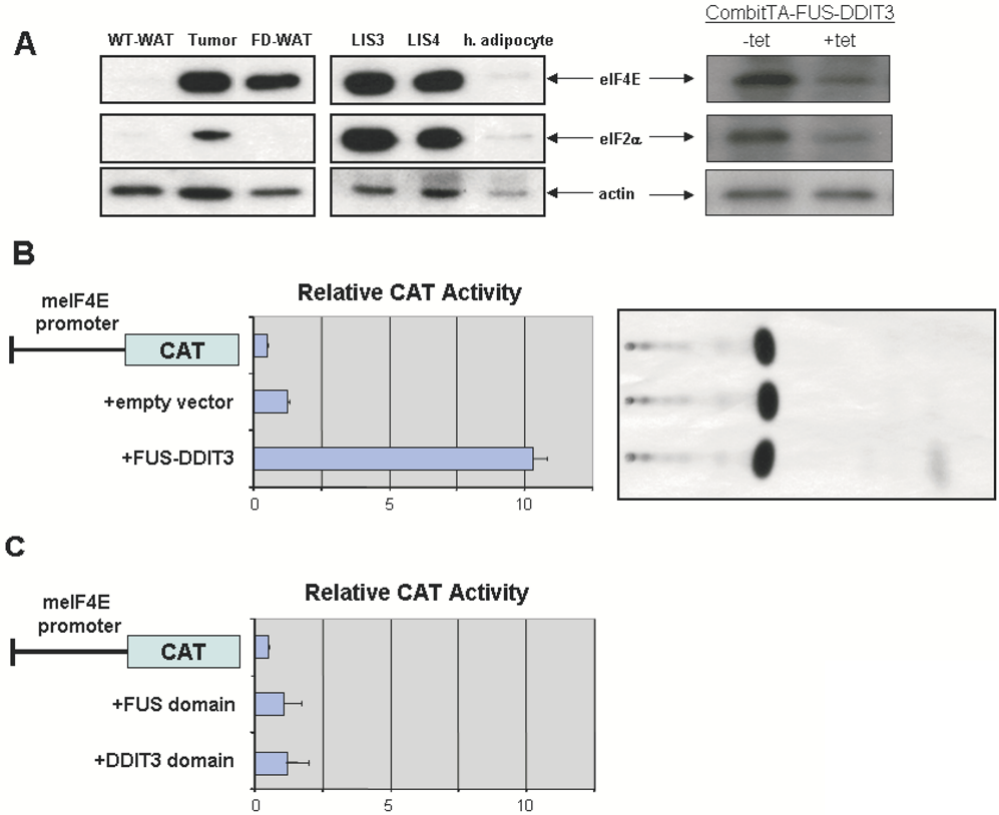
FUS-DDIT3 upregulates eIF2α and eIF4E. (A) Western blot analyses of eIF4E and eIF2α expression in wild-type white adipose tissue (WT-WAT), liposarcoma arisen in FUS-DDIT3 mice (Tumor), normal WAT from FUS-DDIT3 mice (FD-WAT), human liposarcomas cell lines expressing FUS-DDIT3 (LIS-3 and LIS-4), human adipose cells (Zen-bio), and in liposarcoma arisen in CombitTA-FUS-DDIT3 mice in the presence (+tet) or in the absence (−tet) of doxycycline (doxycycline was given at 4 mg/mL for 4 weeks). Cell and tissue extract (10 μg) were resolved in SDS-PAGE gel (10% acrylamide), followed by immunoblotting analysis with anti- eIF4E, anti- eIF2α and anti-actin antibodies. (B) Transactivation of the CAT reporter gene linked to mouse eIF4E promoter by FUS-DDIT3. C3H10T1/2 cells were transiently cotransfected with 1 μg of pm4ECAT (CAT reporter vector containing ∼2.5 kb of the mouse eIF4E promoter) together with 5 μg of pcDNA (empty vector, panel 2) or with 5 μg of pcDNA3-hFUS-DDIT3 (hFUS-DDIT3 expression vector, panel 3). The data represent the fold activation with respect to a sample where reporter alone was transfected (panel 1). Data represent an average obtained from three separated experiments. A representative thin layer chromatograph is shown on the right. (C) Transactivation of the CAT reporter gene linked to mouse eIF4E promoter by FUS and DDIT3 domains of FUS-DDIT3 fusion protein. C3H10T1/2 cells were transiently cotransfected with 1 μg of pm4ECAT together with 5 μg of a vector expressing the FUS domain (panel 2) or with 5 μg of a vector expressing the DDIT3 domain (panel 3). The data represent the fold activation with respect to a sample where reporter alone was transfected (panel 1). Data represent an average obtained from three separated experiments.

Although the inactivation of C/EBPα is not required, itself, for the blockade of adipogenesis in mesenchymal progenitor cells by FUS-DDIT3, however, the C/EBPα isoform ratio shift towards the truncated isoform both in mouse liposarcomas and in human liposarcoma cell lines (**Figure 1B**). It has been previously reported that the control of initiation of translation of C/EBPα and C/EBPβ by the eukaryotic translation initiation factors eIF2 and eIF4E is critical for the behavior of preadipocytes 3T3-L1. Thus, high levels of the eukaryotic translation initiation factors produce a shift towards truncated C/EBP isoforms that, in turn, induce a blockade in the terminal differentiation of 3T3-L1 cells [30]. Thus, we next studied the expression level of eIF2α and eIF4E in FUS-DDIT3-liposarcomas. Analysis by western-blot of protein lysates showed a dramatic overexpression of both eIF4E and eIF2 in liposarcomas arisen both in FUS-DDIT3 mice and human liposarcomas cell lines FUS-DDIT3 positives (**Figure 6A**), explaining the shift towards the truncated p30 isoform of C/EBPα in liposarcomas. Similarly, the impaired expression of both eIF4E and eIF2 in liposarcomas of CombitTA-FUS-DDIT3 mice was normalized following administration of tetracycline (4 gr/L in the drinking water for 2 weeks) (**Figure 6A**), indicating that the fusion protein is directly responsible for the overexpression of both eIF4E and eIF2 in liposarcoma. Moreover, eIF4E was also strongly upregulated in normal WAT of FUS-DDIT3 transgenic mice, suggesting that overexpression of eIF4E may be one of the first events in the initiation of liposarcomas.

Next, we examined whether this upregulation of eIF4E was a direct effect of FUS-DDIT3, or a consequence of the blockade in adipocyte differentiation in liposarcomas, as it has been previously shown that knock out mice for 4E-BP1, a protein that represses cap-dependent translation initiation by sequestering eIF4E, evidenced reduced WAT mass [39]. We examined whether FUS-DDIT3 might be directly involved in the control of the eIF4E expression. In order to address this question, we used a vector containing a CAT gene reporter under the control of ∼2.5 kb proximal promoter region of the murine eIF4E promoter (pm4ECAT) [40]. When U2OS cells were co-transfected with the reported vector along with the FUS-DDIT3 expression vector, there was a specific increae of the CAT activity compared to the activity with the empty vector (**Figure 6B**), demonstrating that FUS-DDIT3 is involved in the upregulation of eIF4E in liposarcomas.

We further investigated which FUS-DDIT3 domain was responsible for the repression of the eIF4E promoter. Using the same system, we proved that neither the co-expression of the domain NH2-FUS nor the co-expression of the DDIT3 domain produced any effect on the transactivation of the eIF4E promoter (**Figure 6C**), indicating that both domains are required for affecting eIF4E expression. These observations establish for the first time the role of FUS-DDIT3 in preventing the development of adipocytic precursors in liposarcoma development.

## Discussion

Current studies support that FUS-DDIT3–associated liposarcomas initiate in uncommitted progenitor cells and generate early adipocytic precursors indicating an important role for *FUS-DDIT3* in the control of early adipocytic development [reviewed in 24]. However, the molecular mechanisms used by FUS-DDIT3 to prevent the development of the adipocytic precursors, leading to the observed buildup of the early precursors in liposarcomas, remain mainly unknown. Here, we have attempted to rigorously unmask the molecular mechanisms associated with this blockade in adipocyte differentiation program of mesenchymal progenitor cells in myxoid liposarcomas harbouring the chromosomal translocation t(12;16)(q13;p11).

**Figure 7:**
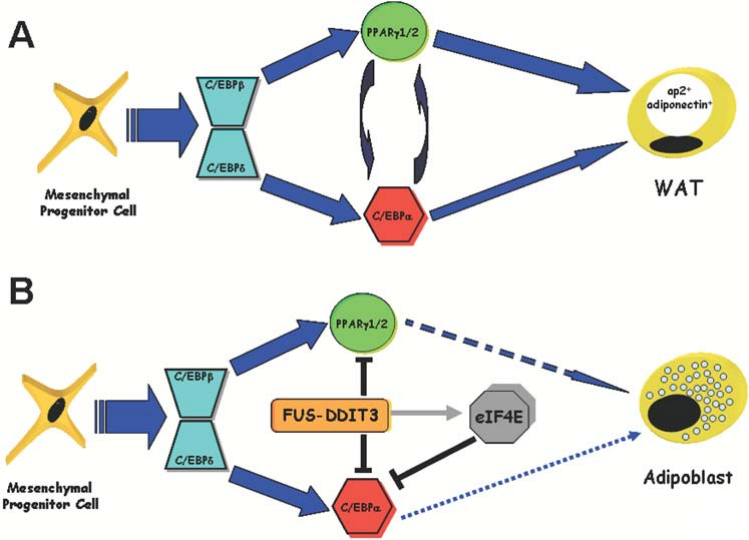
Model for the adipocyte differentiation arrest produced by *FUS-DDIT3* in liposarcoma development. (A) Scheme of the normal differentiation program in mesenchymal progenitor cells. (B) FUS-DDIT3 blocks the adipocyte differentiation program in mesenchymal cell progenitors by interfering with the PPARγ and C/EBPα activities at the transcriptional level. In addition, FUS-DDIT3 induces the expression of eIF4E, that in turns, is able to inactivate the C/EBPα pathway by shifting the normal isoform ratio towards the truncated p30- C/EBPα isoform, which has a negative effect on adipogenesis.

The analysis of the components of the gene regulatory network that controls adipocyte differentiation in liposarcomas developed in FUS-DDIT3 transgenic mice showed a dramatic decreased in the expression levels of the transcription factors involved in the final stages of adipogenesis, such as PPARγ1, PPARγ2 and C/EBPα (**Figure 1A**), while the expression levels of C/EBPδ and C/EBPβ, involved in early stages of adipocyte differentiation, were upregulated (**Figure 1A**). Moreover, we have shown that FUS-DDIT3 interferes with the PPARγ2 and C/EBPα activities at a transcriptional level by repressing their promoter sequences (**Figures 4** **and** **5A**). In agreement with these findings, liposarcomas developed in FUS-DDIT3 transgenic mice also express mature adipocyte markers, such as ap2 or adiponectin, at low levels (**Figure 1C**). These molecular findings corroborate the accumulation of early precursors in liposarcomas developed in FUS-DDIT3 transgenic mice. Interestingly, LIS-3 and LIS-4, two human cells lines derived from liposarcomas expressing *FUS-DDIT3*, showed an almost identical pattern of expression of the components of the gene regulatory network that controls adipocyte differentiation, indicating further that the FUS-DDIT3 transgenic mouse model reproduces accurately the human pathology. Taken together these results strongly support that the expression of the *FUS-DDIT3* oncogene is able to block the adipocyte differentiation program of target mesenchymal progenitor cells interacting with the PPARγ and C/EBPα pathways (**Figure 7**) and contributing to generate a transformed phenotype, similarly to other fusion oncogenes associated to hematopoietic malignances, such as BCR-ABL^p190^ [41] and PML-RARα [42].

C/EBPα and PPARγ are key players in terminal adipocyte differentiation [25]–[27], although both transcription factors have different roles. Thus, while PPARγ is able to induce terminal differentiation in a C/EBPα null background, C/EBPα has no ability to do it in a PPARγ null fibroblast [37], suggesting that PPARγ is the most important transcription factor involved in adipogenesis. Moreover, PPARγ2, which contains an additional 31 amino acids at its amino terminal domain than PPARγ1, has been shown to be the only PPARγ isoform that is able to induce adipogenesis in 3T3-L1 preadipocytes in which PPARγ1 and PPARγ2 were repressed by zinc finger repressor proteins [43]. Moreover, it has been reported that C/EBPα may be required to maintain the expression on PPARγ in adipocytes [38]. Here we have shown that while ectopic expression of PPARγ2 is able to induce final adipogenesis of primary fibroblasts expressing *FUS-DDIT3*, C/EBPα is unable to rescue the impaired adipogenesis of adipocytic precursors expressing FUS-DDIT3, suggesting that the down-regulation of PPARγ2 by FUS-DDIT3 is one of the critical steps in the blockade of adipocyte differentiation in liposarcomas. In addition, we show that the carboxy terminal domain of the fusion protein FUS-DDIT3 is the part of the protein involved in the represion of the PPARγ2 promoter, which is congruent with the *in vitro* role of DDIT3 in adipocyte inhibition of 3T3-L1 cells [15] and liposarcoma development in a model using the HT1080 fibrosarcoma cell line [19]. However, ectopic expression of DDIT3 in a transgenic mouse model did not develop liposarcoma [18] indicating that *in vivo* the cellular environment and the cooperation of both domains of the chimeric protein FUS-DDIT3 play a critical role to induce frank malignancy [24].

The control of the translation initiation of C/EBPβ and C/EBPα mRNAs has been shown to be important in the activity of both transcription factors, as truncated isoforms of them have negative effect on adipogenesis [30]. We also analyzed the ratio of C/EBPβ and C/EBPα isoform expression in liposarcomas and proved that only C/EBPα had a shift towards the truncated isoforms, suggesting that while C/EBPα showed a reduced activity, C/EBPβ had apparently a potential normal activity in liposarcomas. This could be unexpected, but the C/EBPβ activity has been shown to be important in the FUS-DDIT3-mediated interleukin-6 expression [44]. These results suggest FUS-DDIT3 was also interfering with C/EBPα, required to maintain the expression on PPARγ in adipocytes [38]. Interestingly, we found high levels of eIF4E and eIF2α in liposarcomas, two translation initiation factors involved in controlling the ratio of C/EBP isoforms [30]. Moreover, FUS-DDIT3 is able to transactivate the eIF4E promoter suggesting that FUS-DDIT3 is able to interfere with the translational initiation machinery and disrupt the normal adipocyte differentiation program of adipocyte progenitor cells in liposarcomas. The observation that both domains of FUS-DDIT3 are required to regulate eIF4E expression provides the first molecular evidence that the FUS component of the fusion protein is required not only for transformation but also influences the phenotype of the tumor cells. The eIF4E is frequently overexpressed in human cancers in relation to disease progression and drives cellular transformation [reviewed in 45]. In this sense, eIF4E was also strongly upregulated in normal adipose tissue of FUS-DDIT3 transgenic mice, suggesting that overexpression of eIF4E may be one of the primary events in the initiation of liposarcomas.

In conclusion, we demonstrate that FUS-DDIT3 is able, itself, to impede the normal adipogenesis in mesenchymal progenitor cell contributing to achieve a transformed phenotype by blocking the adipocyte differentiation program. In order to achieve this, FUS-DDIT3 blocks the activity of the most vital adipogenic transcription factors: C/EBPα and PPARγ. In addition, we show that this blockade is produced at two levels. First of all, FUS-DDIT3 represses both C/EBPα and PPARγ2 promoters, reducing the expression of both transcription factors. Additionally, the chimeric protein, obstructing the normal translational initiation activity of at least eIF4E, is able to shift towards truncated isoform the expression of C/EBPα, reducing its activity and contributing to attenuate the positive feedback loop between C/EBPα and PPARγ that finally results in the expression of mature adipocyte markers such us ap2 or adiponectin (**Figure 7**). These results will help to develop a strategy that would form the basis for improved therapy in human liposarcomas.
